# ML-FSID-FIS: A Multi-Level Feature Selection and Fuzzy Inference System for Intrusion Detection in IoMT

**DOI:** 10.3390/s26082501

**Published:** 2026-04-18

**Authors:** Ghaida Balhareth, Mohammad Ilyas, Basmh Alkanjr

**Affiliations:** 1 Department of Electrical Engineering & Computer Science, Florida Atlantic University, 777 Glades Road, Boca Raton, FL 33431, USA; balkanjr2019@fau.edu; 2 Department of Information Systems, College of Computer Science and Information System, Najran University, Najran 61441, Saudi Arabia; 3 Department of Computer Science, College of Computer and Information Science, Jouf University, Sakaka 72388, Saudi Arabia

**Keywords:** Intrusion Detection System (IDS), machine learning, fuzzy logic, feature selection, fuzzy inference system (FIS), IoMT

## Abstract

The Internet of Medical Things (IoMT) is becoming a vital part of modern healthcare, enabling ongoing patient monitoring and remote diagnosis. However, as more devices connect to the internet, healthcare systems become more vulnerable to serious security issues such as unauthorized access, patient data manipulation, and Man-in-the-Middle attacks. Conventional Intrusion Detection Systems (IDSs) often struggle with the unclear and uncertain characteristics of IoMT traffic, which leads to reduced detection accuracy and increased false alarms. To address these challenges, this paper proposes ML-FSID-FIS, a multi-level feature selection-based Intrusion Detection System that employs a fuzzy inference system (FIS) for classification in IoMT networks. The model combines multiple feature selection techniques into a three-stage multi-level feature selection strategy to improve detection efficiency and strengthen the security of IoMT networks. In the first stage, four feature selection techniques—Random Forest, XGBoost, ReliefF, and Mutual Information—are applied to identify the most relevant features. In the second stage, a frequency-based consensus strategy is utilized to extract consistently selected features from the four top-ranked sets. In the third stage, an ensemble refinement using bagging-based ranking is employed to rank the remaining features, resulting in the selection of the top five features. From these, three candidate 3-feature groups are formed and evaluated, and the best-performing group is selected as the final input set for the fuzzy logic classifier. The FIS produces a continuous risk score that is mapped to a binary decision using a validation-selected threshold. When the proposed method was tested on the WUSTL-EHMS-2020 dataset and compared with other recent work using the same dataset, it showed strong detection performance while maintaining a very low false positive rate of 0.3%. This study is distinguished by its integrated design, which combines a three-stage multi-level feature selection strategy with fuzzy logic-based intrusion classification to improve feature efficiency and support interpretable intrusion detection in IoMT.

## 1. Introduction

The Internet of Medical Things (IoMT) has become a transformative development in today’s healthcare system. It connects doctors, medical workers, and patients in a seamless way. This technology helps monitor patients as they go about their day, makes diagnosing diseases more accurate, and enables doctors to give better treatment. It does this by sharing important medical information quickly and efficiently [1]. However, as healthcare becomes more connected through digital tools, new security issues are appearing. Medical devices that are linked to the internet are more at risk from cyber threats such as stolen data, unauthorized access, and attacks that could put patients in danger or compromise the accuracy of their medical information [2].

Moreover, many industry analysts also anticipate that the IoMT market will grow rapidly until 2030. For instance, Grand View Research expects the market to reach $658.57 billion by 2030, growing at a rate of about 18.2% each year from 2025 to 2030 [3]. This rapid growth highlights the importance of having robust security systems to protect IoMT networks from new and changing threats. At the same time, the growing market presents more opportunities for cyberattacks from malicious actors, which puts sensitive medical information and patient safety at higher risk.

IoMT devices handle highly sensitive medical data, and if this data is compromised, it can lead to life-threatening situations [4]. Attacks in IoMT environments can be significantly more serious than those in general IoT systems. For example, if someone injects false information into a smart thermostat in a home, it might just cause some discomfort, such as the temperature not being right. However, if the same attack were to occur on a medical device, such as a thermometer used to monitor a patient’s health, it could result in the wrong medication being administered. This scenario can cause serious health problems or even death, demonstrating that the risks associated with IoMT are much greater than in other IoT contexts.

Man-in-the-Middle (MitM) attacks are one of the most serious threats to IoMT networks. In this attack, an attacker secretly intercepts communication between devices and can change or add false information. One common type is spoofing, where the attacker pretends to be a trusted server or device to trick the system [5]. In serious situations, this kind of interference can put patients at risk and disrupt the operation of medical equipment.

**Figure 1:**
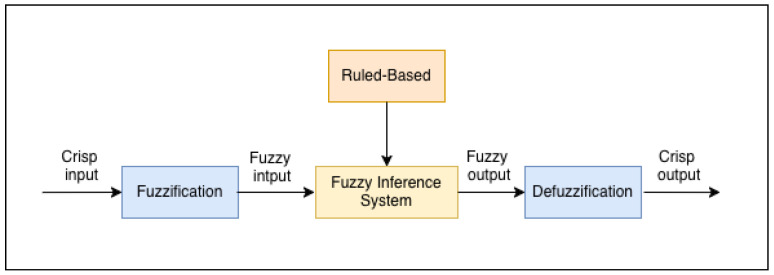
Generic structure of a fuzzy inference system (FIS).

Intrusion Detection Systems (IDSs) are commonly used to detect and prevent malicious network activity. However, many IDS systems still struggle to deal with advanced attacks and may not perform consistently. One reason is that many IDSs rely on strict normal-or-attack rules, which can cause issues when trying to handle uncertain or unclear situations. These strict rules can lead to misclassifications, because some intruders cannot be accurately captured using a crisp threshold. Fuzzy logic, based on Zadeh’s fuzzy set theory [6], helps with this issue by handling uncertainty and imprecision. It uses linguistic terms (e.g., low, high) and sets up if–then rules that represent experts’ knowledge, making a graded risk level instead of only a binary decision. This is especially useful in an IoMT environment, where data can be noisy and incomplete [7,8]. Figure 1 shows the general structure of a fuzzy inference system, where crisp inputs are fuzzified, then evaluated using a rule-based inference engine, and then defuzzified to generate a crisp output.

Based on these foundations, this study proposes ML-FSID-FIS, an Intrusion Detection System for IoMT networks that combines multi-level feature selection with a fuzzy inference system. The novelty of the proposed work lies not in introducing an entirely new standalone learning algorithm, but in integrating complementary components into a unified IoMT intrusion detection framework. In particular, the proposed model combines a three-stage multi-level feature selection process, a compact zero-order Sugeno fuzzy inference classifier, and an evaluation with an emphasis on low false positive behavior in a safety-critical IoMT setting. This combination is intended to improve feature efficiency, maintain interpretability, and support practical intrusion detection under uncertain IoMT traffic conditions.

### 1.1. Contributions

This paper presents a practical and interpretable intrusion detection approach for IoMT networks. The main contributions are:

- This study presents an IoMT intrusion detection framework on the WUSTL-EHMS-2020 dataset that combines a three-stage multi-level feature selection process with a compact zero-order Sugeno fuzzy inference system.
- We develop a multi-level feature selection strategy that applies four different methods—Random Forest, XGBoost, ReliefF, and Mutual Information—to rank features and reduce redundancy. Using multiple selectors helps capture different views of feature importance and reduces dependence on any single method.
- We combine the selected feature sets using a frequency-based consensus strategy that retains consistently selected features across the four methods. We then apply a bagging-based ranking procedure to refine this subset and retain the top five features. From these, we form three candidate 3-feature groups and select the best-performing group as the final FIS input set, improving efficiency while maintaining detection capability.
- We evaluate ML-FSID-FIS using a class-stratified train–validation–test split and standard metrics, including accuracy, Precision, F1-score, FPR, and ROC/AUC. We also analyze Recall versus FPR to study performance under low-false alarm requirements in safety-critical IoMT environments.
- We compare the proposed model with recent studies on detecting intrusions in IoMT systems that use the WUSTL-EHMS-2020 dataset, focusing on detection performance, false alarm behavior, feature efficiency, and interpretability.

### 1.2. Organization of the Paper

The remainder of this paper is organized as follows: Section 2 reviews related works on intrusion detection in IoMT environments. Section 3 presents the proposed methodology, including dataset description, data preprocessing, multi-level feature selection, and the classification phase. Section 4 describes the implementation environment and the tools used. Section 5 reports the evaluation metrics and the experimental results. Section 6 compares the proposed work with recent state-of-the-art methods. Finally, Section 7 concludes our paper.

## 2. Related Works

Due to the growing attack surface and heightened safety requirements in the medical sector, research on intrusion detection for the IoMT has expanded rapidly in recent years. This section summarizes representative methodologies, datasets, and results, providing context for the strengths and limitations of current IoMT intrusion detection studies.

In [9], the authors present XMeDNN, a deep neural network (DNN)-based Intrusion Detection System (IDS) made for Internet of Medical Things environments. They use the WUSTL-EHMS-2020 dataset to train and test a multi-layer perceptron model, which achieves an accuracy of 96.87%, an F1-score of 0.968, and a false positive rate of 3.12%. They also use SHapley Additive Explanations (SHAPs) to explain how each feature contributes to the model’s predictions. In a similar study, ref. [10] addresses the “black-box” issue of deep learning by adding SHAP-based explanations to an edge-assisted deep learning IDS. Their work also uses Particle Swarm Optimization (PSO) to improve the feature engineering process and improve detection efficiency.

Several studies have investigated tree-based and ensemble learning methods for IoMT intrusion detection. In [11], the authors suggest a lightweight IDS that combines Random Forest with robust scaling and SMOTE to address class imbalance and improve minority-class detection. They also apply hyperparameter tuning to optimize the model for distinguishing normal traffic from attack traffic. Their approach achieves better performance than baseline models such as logistic regression, decision trees, and extra trees.

In [12], the authors proposed an Intrusion Detection System for IoMT by combining PSO for feature selection with AdaBoost to reduce bias during classification. Upon evaluation, the model shows 98.7% accuracy and 96.7% Recall, which supports the benefit of combining feature optimization with ensemble learning. The framework presented in [13] integrates Recursive Feature Elimination (RFE) and Ridge regression as well as machine learning and deep learning models. RFE and Ridge regression are used to find the most relevant features, after which various classifiers are trained to distinguish between normal and attack traffic. On the WUSTL-EHMS dataset, the RFE-based Decision Tree version performs best among the evaluated methods.

Other studies aim to improve intrusion efficacy via meta-learning or explainability. In [14], the authors introduced ME-IDS, a framework based on meta-learning that helps enhance ensemble-based IDS systems used in IoMT environments. They tested ME-IDS against other systems like Stack–IDS, DIS-IoT (a distributed IDS for IoT), and EDL-IDS (an ensemble deep learning IDS), which frequently show good detection accuracy across a feature subset of 5 to 45 features, thus suggesting robustness to feature dimensionality. They used the WUSTL-EHMS-2020 dataset for testing, but the study did not give detailed information about how the data was labeled, so it is unclear if the classes were balanced in their experiments. In [15], they used SHAP-enhanced ensemble IDS to clarify feature significance across various datasets, showing the importance of model interpretability, especially in a security-sensitive IoMT environment.

In addition to designing models, some studies focus on architectural and deployment concerns. The framework in [16] utilizes fog computing to enhance data processing efficiency in the IoMT. It works in two steps: first, it selects incoming streams, followed by a subsequent phase that categorizes causes of stress. When testing different models like KNN, SVM, Bagged Tree, and ANN, the ANN performed best with an F1-score of 99.97%. By sending priority tasks to the fog layer, this system lowers delays and uses resources more efficiently for real-time healthcare. In [17], a meta-IDS is introduced that combines a Linear SVM, a Convolutional SVM, and the CatEmb method. The deep learning models get very high accuracy: Linear SVM at 99.78%, ConvSVM at 99.98%, and CatEmb at 99.84%. The meta-IDS exhibits resilience and minimal misclassification rates across various datasets, like WUSTL-EHMS-2020, IoTID20, and WUSTL-IIoT-2021. It detects anomalies at rates from 99.47% to 99.99%, and uses signature-based detection at rates from 99.57% to 99.99%.

Deep learning-focused IDSs have been studied for use in IoMT and similar areas. A study in [18] suggests a deep learning-based IDS that tackles data imbalance in the WUSTL-EHMS-2020 dataset by using cost-sensitive learning, instead of data preprocessing or augmentation methods. This model uses a global attention mechanism to connect convolutional neural networks (CNNs) and long short-term memory (LSTM) layers, allowing the model to collect both spatial and temporal patterns from network traffic and patient health information. The findings indicate enhanced performance compared to several prior IoMT IDS methodologies. Another study in [19] introduces an Intrusion Detection System for the Internet of Health Things (IoHT) that combines dynamic autoencoders with multi-class support vector machines (SVMs). Autoencoders help feature extraction and optimization in real time, while SVMs identify attack signatures and assign them to different levels of severity, helping with more detailed and precise responses. The study in [20] presents a blended deep learning model for IoMT that merges CNN and LSTM structures to identify attacks in healthcare networks, utilizing CNNs for extracting local features and LSTMs for temporal modeling.

Overall, current research on IoMT IDSs indicates that high detection performance can be achieved using deep neural architectures, ensembles, and advanced feature engineering. Many approaches, however, depend on an extensive feature set, complex deep models, or fail to systematically report false positive rates and address class imbalance, complicating their implementation in resource-constrained and safety-critical IoMT environments. These gaps motivate the development of ML-FSID-FIS, which combines multi-level feature selection, a compact fuzzy inference classifier, and explicit evaluation of false alarm behavior on the WUSTL-EHMS-2020 dataset. Unlike many prior studies that rely mainly on deep architectures, larger feature sets, or less interpretable designs, the proposed framework emphasizes a compact feature subset, interpretable fuzzy reasoning, and low-false-alarm performance in an IoMT setting.

## 3. Methodology

This study develops and assesses ML-FSID-FIS, a multi-level feature selection and fuzzy inference-based intrusion detection model for IoMT networks. The proposed workflow consists of three phases: data preprocessing, multi-level feature selection, and classification. During the data preprocessing phase, the raw WUSTL-EHMS-2020 data are cleaned, normalized, missing values are addressed, class imbalance is handled, and records are turned into a format suitable for machine learning. These steps enhance data integrity and ensure unified, consistent inputs for further analysis.

In the feature selection phase, a multi-level approach is used to minimize redundancy, enhance interpretability, and improve classification performance. First, four methods—Random Forest, Mutual Information, ReliefF, and XGBoost—are utilized to choose the best features based on their significance to the target label. Second, the results from these methods are combined to preserve features that are consistently emphasized across methods by applying a frequency-based consensus. Third, a bagging-based ensemble ranking further refines the consensus feature subset and retains the top five features. From these five features, three candidate 3-feature groups are formed and evaluated, and the best-performing group is selected as the final input set for classification.

**Figure 2:**
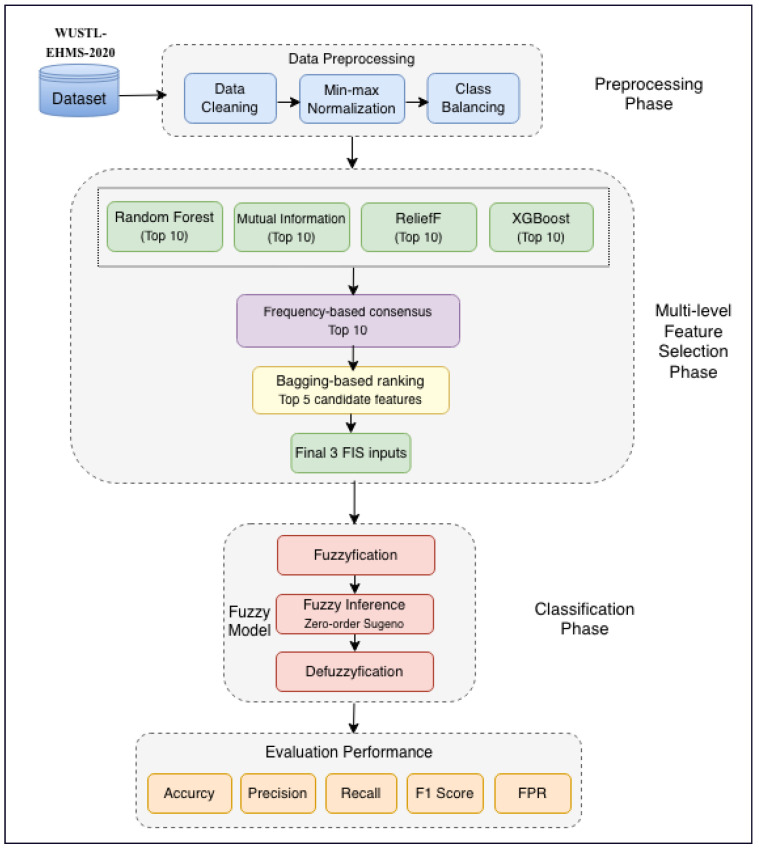
Architecture of the proposed ML-FSID-FIS model.

In the classification phase, a fuzzy inference system (FIS) categorizes each traffic event as either normal or an attack. The fuzzy logic classifier is designed to manage uncertainty and overlapping patterns in IoMT traffic, thus minimizing misclassification. The following sections explain each phase in detail. Figure 2 summarizes the overall workflow of ML-FSID-FIS.

### 3.1. Overview of the Proposed Model

IoMT networks suffer major security issues, especially in identifying attacks that can harm important medical information and put patients at risk. To address this, we propose an ML-FSID-FIS, a multi-level feature selection-based Intrusion Detection System that uses a fuzzy inference system as a classifier. The methodology consists of three phases: first, data preprocessing; second, multi-level feature selection; and third, a fuzzy inference system for classification.

#### 3.1.1. Dataset Description

The WUSTL-EHMS-2020 dataset is a comprehensive dataset used for assessing IDSs in IoMT environments. It was collected from a real healthcare monitoring testbed that uses wearable medical devices, a network gateway, and a software-defined network controller to simulate realistic IoMT data transmission. This dataset was selected because it provides a healthcare-oriented IoMT benchmark that includes both network flow and biometric information, making it suitable for evaluating intrusion detection in a realistic medical monitoring setting. The dataset includes examples of three main types of cyber threats: MITM attacks, in which attackers hack and modify communications; spoofing attacks, in which an attacker impersonates legitimate devices to obtain unauthorized access; and data injection attacks, which manipulate transmitted data to make it unreliable.

**Figure 3:**
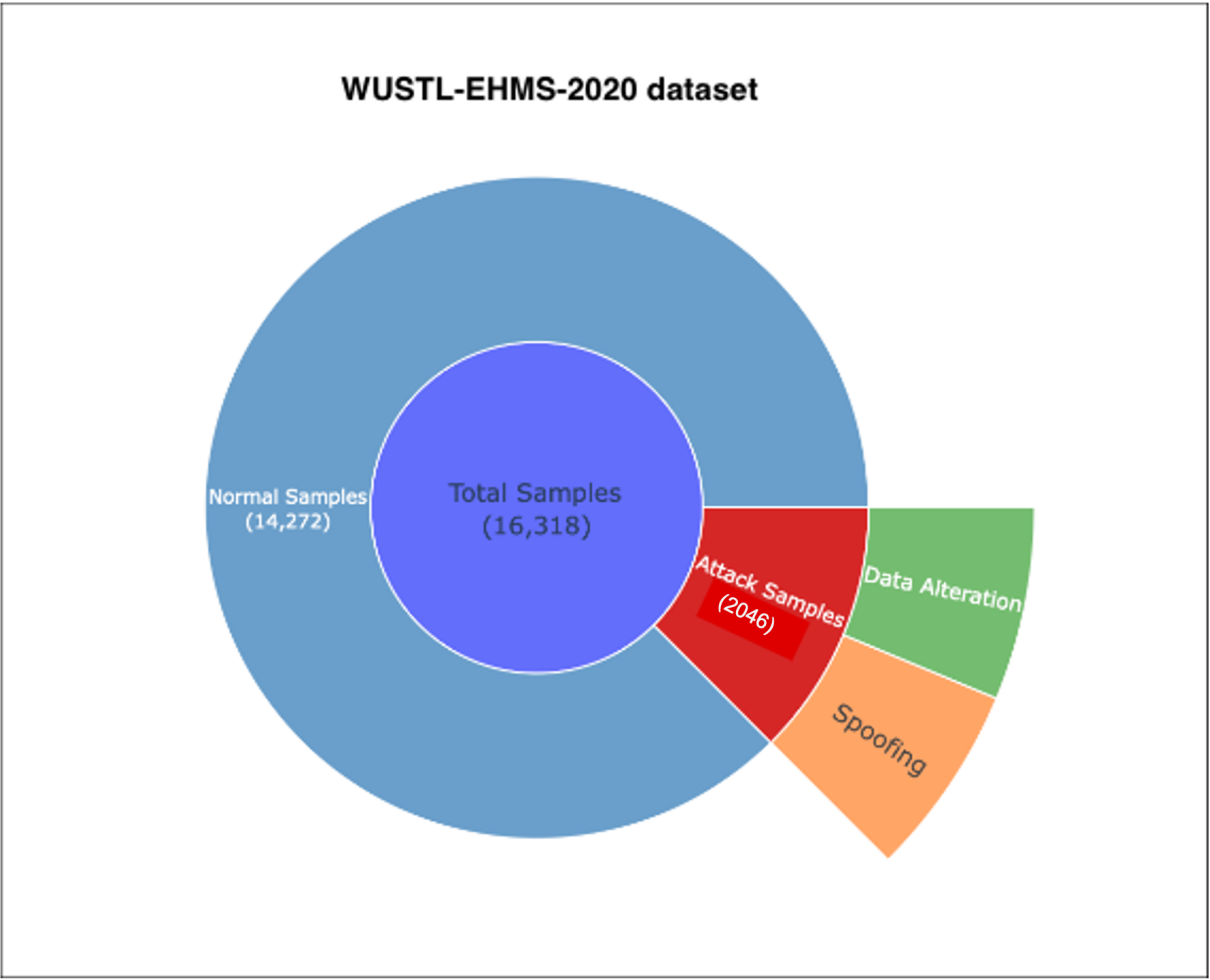
Distribution of normal and attack samples in the WUSTL-EHMS-2020 dataset.

The dataset consists of 44 attributes in total: 43 input features (network-related and biometric) and 1 binary label that distinguishes attack instances (1) from normal traffic (0). The input attributes include a combination of network flow metrics collected from traffic monitoring tools and biometric measurements obtained from medical sensors. Table 1 presents representative examples of these features along with their brief descriptions. By combining flow-level and biometric information, WUSTL-EHMS-2020 offers a realistic evaluation framework for IDS models in IoMT, supporting the detection of both network anomalies and abnormal physiological patterns. The distribution of normal and attack samples is illustrated in Figure 3.

#### 3.1.2. Data Preprocessing Phase

Efficient data preparation is critical for accurate intrusion detection in IoMT. The handling of missing values, feature scaling, and the resolution of class imbalance are all determined by proper preprocessing. These steps help improve model performance, ensure data quality, and support robust machine learning analysis [21]. In this work, three primary preprocessing steps are utilized:

- **Data cleaning:** To ensure dataset integrity, missing values and duplicate records are addressed. Duplicate entries are identified through a comparative analysis of rows based on key attributes to guarantee the uniqueness of each observation, hence avoiding bias or distortion from repeated instances. Host-specific features, including MAC source and destination addresses as well as IP source and destination addresses, are excluded due to their lack of contribution to intrusion detection in this context [15]. In addition, since one of the feature selection techniques (Mutual Information) requires non-negative, non-categorical inputs, the categorical features dir and flag are also removed [22]. Overall, these cleaning procedures result in the removal of six input features, leaving 37 input features plus the label, 38 attributes in total available for subsequent analysis.
- **Normalization:** To improve the stability and efficiency of training, numerical features are normalized using min–max scaling [23]. Each attribute is rescaled to the range [0,1], preserving relative relationships among data points while reducing the impact of differing feature magnitudes. This guarantees that all features contribute equally during model training and prevents features with larger numeric ranges from emphasizing the learning process.
- **Balancing:** The original dataset exhibits class imbalance between normal and attack traffic. To mitigate this, the Synthetic Minority Over-sampling Technique (SMOTE) is applied to generate synthetic samples of the minority class. This yields a more balanced dataset, reduces bias toward the majority class, and improves the model’s ability to accurately detect intrusions—an essential requirement for an effective IoMT IDS.

#### 3.1.3. Feature Selection Phase

In order to improve generalization, decrease overfitting, and minimize computational cost, feature selection aims to reduce dimensionality by determining a subset of the most useful features for classification [24]. Feature selection plays a critical role in IDS for the IoMT by helping the model focus on the key attributes that are critical for identifying attacks, while eliminating irrelevant or noisy variables. In the first stage of the proposed multi-level feature selection, four methods are applied independently to rank feature importance: Random Forest, Mutual Information, ReliefF, and XGBoost. Each method produces an importance score for every feature, from which we select the top 10 features according to their own ranking. The following subsections briefly describe these techniques.

- **Mutual Information (MI).** It is a statistical way to measure dependency, which estimates how much knowing one variable reduces uncertainty about another. In our case, MI measures how strongly it is related to the class label, making it appropriate for identifying features that transmit useful information for separating normal from attack traffic in an IoMT network.
- **Random Forest (RF).** This ensemble of decision trees is trained on different subsets of samples and features [25]. Feature importance is usually calculated by looking at the average reduction in impurity or prediction error that each feature contributes across all the trees it is part of. This enables RF to emphasize both strongly and slightly significant features, while staying efficient and requiring minimal parameter adjustment.
- **XGBoost.** This gradient-boosting method builds a sequence of decision trees, where each new tree focuses on the residual errors of the previous ones [26]. Feature importance is inferred from how frequently and how effectively features contribute to reducing the loss function across the boosted trees, capturing complex, non-linear interactions between features and the target.
- **ReliefF.** The Relief-based technique adjusts feature weights iteratively using *k* nearest neighbors from each class, extending the original Relief method. By analyzing feature differences across nearby instances of the same and different classes, it identifies properties that are consistent within classes yet discriminative across classes. ReliefF works well even when there is noise or missing data, and it can handle larger datasets effectively, which makes it a suitable fit for our situation [27].

Table 2, Table 3, Table 4 and Table 5 list the top 10 features selected by each method. Using multiple feature selection methods allows us to capture different notions of feature relevance. RF focuses on features that enhance the accuracy of tree-based predictions, MI identifies statistical dependencies related to the target, XGBoost targets attributes that minimize the loss in boosted ensembles, and ReliefF identifies features that clearly separate nearby instances across classes while maintaining resilience to noise. This diversity reduces the risk that important features are missed due to the bias of any single method.

In the second stage, we combine the four top 10 lists to find features that are consistently preferred by different selectors. Instead of requiring strict overlap, we adopt a frequency-based consensus strategy that prioritizes features that appear most often across the four rankings. Specifically, we aggregate the four top 10 lists, calculate the selection frequency of each feature, and retain the top 10 features with the highest frequencies as the consensus set. The resulting consensus feature subset is summarized in Table 6.

**Figure 4:**
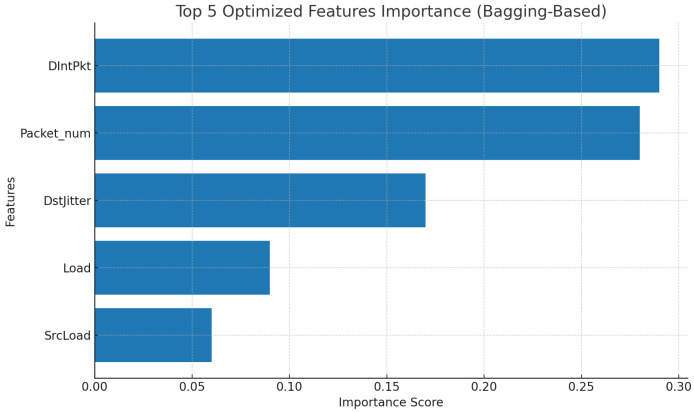
Bagging-based importance ranking of the consensus feature subset.

In the third stage, we identify the most appropriate features from this common set by a bagging-based ranking method. Bagging is an ensemble technique where multiple models are trained on bootstrap samples from the training data, and their results are combined to reduce the chance of overfitting and improve stability [28]. We apply the bagging approach only on the training data to obtain a reliable, leakage-free ranking of the consensus feature subset. The resulting importance ranking is shown in Figure 4, from which we retain the top-ranked five features (DIntPkt, Packet_num, DstJitter, Load, SrcLoad) as the final output of the multi-level feature selection process. Overall, this phase reduces the number of input features from 37 to 5, discarding the rest.

These five features form the basis for the three candidate 3-input groups (A, B, and C), from which the best-performing group is selected for the FIS classification phase described in the next subsection. Rather than exhaustively evaluating all ten possible 3-feature combinations, this study selected three representative candidate groups from the top five ranked features to provide a controlled comparison while keeping the fuzzy inference system compact and interpretable. In particular, the selected groups were designed to examine the effect of the third feature while retaining the strongest shared features across the candidate sets. Therefore, Group B is identified as the best among the evaluated candidate groups.

#### 3.1.4. Classification Phase

**Figure 5:**
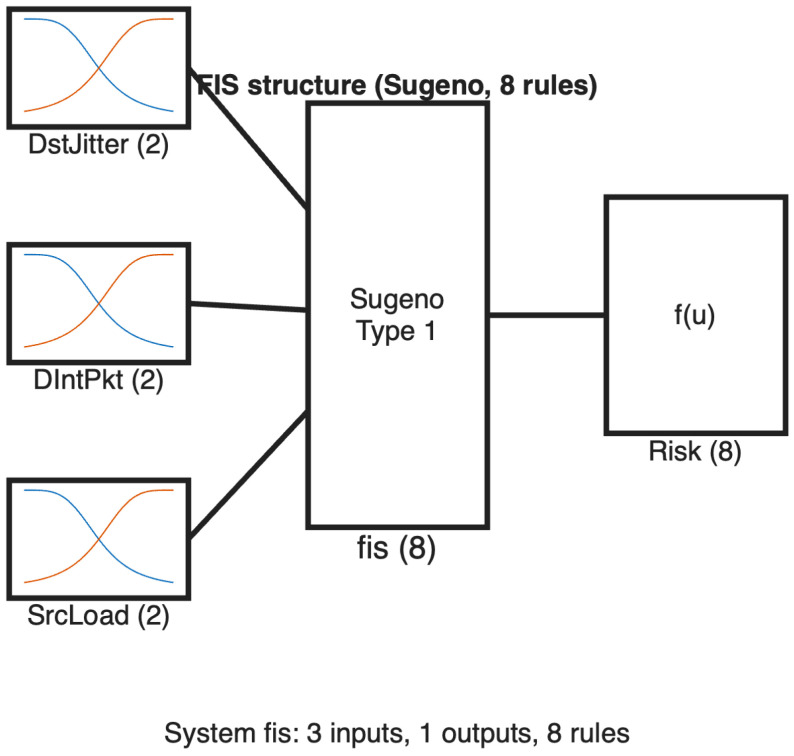
Structure of the ML-FSID-FIS fuzzy classifier with three inputs, eight Sugeno rules, and a single risk score *y*.

After the multi-level feature selection phase, the top three features from the bagging-ranked top five are used as inputs to a zero-order Sugeno fuzzy inference system (FIS), as illustrated in Figure 5. We utilize a Sugeno-type FIS due to its computing efficiency and compatibility with optimization and adaptive methods. In a zero-order Sugeno system, each rule has a constant output value [29]. A fuzzy inference system translates crisp inputs into outputs by modeling variables as fuzzy sets and integrating them via a rule base. This helps the classifier handle uncertainty and overlapping traffic patterns, while keeping the system simple and interpretable.

In our model, each of the three input features is divided into two linguistic terms (*Low* and *High*), using a generalized bell membership function textttgbellmf. The fuzzy rule base increases exponentially with two terms per input, calculated as $2^{K}$; for K=3 inputs, this results in $2^{3}=8$ rules. This small rule base keeps inference lightweight and the model easy to audit without materially sacrificing performance:

1. IF DstJitter is Low AND DIntPkt is Low AND SrcLoad is Low, THEN the rule contributes to the final risk score through its constant Sugeno output.
2. IF DstJitter is Low AND DIntPkt is Low AND SrcLoad is High, THEN the rule contributes to the final risk score through its constant Sugeno output.
3. IF DstJitter is Low AND DIntPkt is High AND SrcLoad is Low, THEN the rule contributes to the final risk score through its constant Sugeno output.
4. IF DstJitter is Low AND DIntPkt is High AND SrcLoad is High, THEN the rule contributes to the final risk score through its constant Sugeno output.
5. IF DstJitter is High AND DIntPkt is Low AND SrcLoad is Low, THEN the rule contributes to the final risk score through its constant Sugeno output.
6. IF DstJitter is High AND DIntPkt is Low AND SrcLoad is High, THEN the rule contributes to the final risk score through its constant Sugeno output.
7. IF DstJitter is High AND DIntPkt is High AND SrcLoad is Low, THEN the rule contributes to the final risk score through its constant Sugeno output.
8. IF DstJitter is High AND DIntPkt is High AND SrcLoad is High, THEN the rule contributes to the final risk score through its constant Sugeno output.

The antecedent part of the rule base is determined by the fuzzy partition of the three selected input features rather than by manually specifying unrelated rules one by one. Since each input is represented by two linguistic terms (*Low* and *High*), all possible combinations generate $2^{3}=8$ rules. Therefore, the rule structure follows directly from the chosen input variables and membership partitions, which improves consistency and reproducibility.

In the zero-order Sugeno FIS, each rule is associated with a constant output, and the final risk score is obtained by combining the active rules according to their firing strengths. For example, one rule can be written as follows: IF DstJitter is High AND DIntPkt is Low AND SrcLoad is High, THEN the rule contributes to the final risk score through its constant Sugeno output.

The FIS works through five computational steps: (i) fuzzification—turns each crisp inputs into membership degrees; (ii) an inference engine—to combine these degrees with a minimum T-norm to find rule firing strengths; (iii) normalization—rescales these firing strengths before evaluating the results; (iv) consequents—with constant outputs for each rule (zero-order Sugeno); and (v) output aggregation—calculates a Sugeno weighted average of the consequents. This procedure produces a continuous risk score y∈[0,1], which is converted to a binary class label using a decision threshold τ selected on the validation split.

For the experimental protocol, the dataset is stratified by class into training 70%, validation 15%, and test 15% splits. The FIS is fit on the training split; the validation split is used exclusively to select τ and verify settings; and the held-out test split provides an unbiased estimate of generalization.

(i) Fuzzification

This level converts each crisp input value into degrees of membership in predefined linguistic terms (*Low* and *High*). In our system, each input feature is described by generalized bell-shaped membership functions (gbellmf), which provide smooth transitions between terms and are efficient to compute. For a scalar input *x*, the membership degree is computed using

(1) $$\mu \left(x;a,b,c\right)\;=\;\frac{1}{1+{\left|\frac{x-c}{a}\right|}^{2b}},$$

where *c* controls the center, a>0 sets the width, and b>0 adjusts the slope. Compared to four-parameter trapezoidal functions, this three-parameter form simplifies tuning while still enabling fuzzy sets to fit the ranges of actual data [30]. In practical terms, these three parameters determine the position and shape of each fuzzy set over the normalized input range. The parameter *c* places the fuzzy set around a representative region of the feature values, *a* controls how broadly the set spans that region, and *b* determines how gradually or sharply the transition occurs between low and high membership. The generalized bell membership function was adopted in this study because it provides smooth and flexible transitions between linguistic terms while remaining computationally efficient for a compact Sugeno FIS. Each selected feature (DstJitter, DIntPkt, SrcLoad) is mapped to two overlapping terms (*Low*/*High*). The resulting membership degrees are passed to the rule layer, allowing the FIS to reason with partial evidence rather than hard thresholds.

(ii) Inference engine

As described in the previous step, the three inputs with two terms each give rise to $2^{3}=8$ fuzzy rules. In the inference layer, the antecedent degrees for each rule are combined with a T-norm to produce a firing strength. Using the minimum T-norm, for rule *r* with antecedents $A_{1}^{\left(r\right)},A_{2}^{\left(r\right)},A_{3}^{\left(r\right)}$ and input $x=(x_{1},x_{2},x_{3})$, the firing strength $\alpha_{r}$ is calculated using

(2) $$\alpha_{r}\;=\;\min\;\left\{\mu_{A_{1}^{\left(r\right)}}\left(x_{1}\right),\;\mu_{A_{2}^{\left(r\right)}}\left(x_{2}\right),\;\mu_{A_{3}^{\left(r\right)}}\left(x_{3}\right)\right\}.$$

Here $\alpha_{r}\in \left[0,1\right]$ measures how well the current inputs satisfy the rule’s conditions: better agreement leads to higher $\alpha_{r}$. The eight firing strengths are then forwarded to the normalization and consequent layers.

(iii) Normalization

After inference, each of the N=8 rules has an associated firing strength $w_{i}\in \left[0,1\right]$. These are converted to normalized weights ${\bar{w}}_{i}$ that sum to one, computed as

(3) $${\bar{w}}_{i}\;=\;\left\{\begin{array}{cc} \frac{w_{i}}{\sum_{j=1}^{N}w_{j}}, & \mathrm{if}\;\sum_{j=1}^{N}w_{j}>0,\\ \frac{1}{N}, & \mathrm{if}\;\sum_{j=1}^{N}w_{j}=0\;\;(\mathrm{all}\;\mathrm{rules}\;\mathrm{inactive}). \end{array}\right.$$

This step maintains the proportional support of the active rules, prevents any single rule from overwhelming by magnitude, and avoids division by zero when all rules are inactive. The normalized weights $\left\{{\bar{w}}_{i}\right\}$ are then used when combining the outputs.

(iv) Consequents

In the next layer, each rule generates a constant output $c_{i}$ (zero-order Sugeno). These constant consequents are associated with the Sugeno rules during model construction and represent the contribution of each rule to the final risk score when its condition is activated. The effect of a rule on the final choice is only determined by its firing strength and normalized weight, as the consequents are not dependent on the present input values. This improves interpretability and helps prevent overfitting.

(v) Output aggregation

In the final step, Sugeno defuzzification is used to combine the normalized weights and rule consequents into a crisp risk score via weighted averaging. The output score *y* is computed as

(4) $$y\;=\;\sum_{i=1}^{N}{\bar{w}}_{i}\;c_{i},$$

where $\sum_{i=1}^{N}{\bar{w}}_{i}=1$. Rules that match the input more strongly (higher ${\bar{w}}_{i}$) contribute more to the final score. The scalar output y∈[0,1] is then mapped to a binary label using the validation-selected threshold τ, ensuring a consistent operating point across all experiments.

### 3.2. Deployment of ML-FSID-FIS in IoMT Networks

In this environment, various medical and wearable devices transmit patient data to hospital systems through a gateway or edge device. While encryption can protect payload confidentiality during transmission, IoMT implementations are still vulnerable to attacks that target the communication pathway or nearby network services (e.g., spoofing, injection, and manipulation). These attacks are particularly critical in healthcare, as manipulated or malicious traffic can disrupt operational processes or lead to incorrect medical decisions.

To address these concerns without modifying resource-constrained devices, ML-FSID-FIS is deployed at the gateway level, where traffic from multiple medical devices is aggregated before reaching the hospital server. At this point, network flow features are extracted from traffic data and provided to ML-FSID-FIS for intrusion detection. This gateway-level deployment enables centralized monitoring while preserving device constraints. Furthermore, since the model relies on flow-level features rather than payload inspection, it does not require decryption and avoids access to sensitive patient data.

## 4. Environment Tools

The proposed ML-FSID-FIS model was developed and tested using Python 3.10 along with these libraries: scikit-learn 1.5, NumPy 1.26, Pandas 2.1, Matplotlib 3.9, and Seaborn 0.13. The FIS part of the model was built using a Python tool designed for fuzzy logic. The experimental evaluation was done on a workstation with an Intel® Core™ i7 processor, 16 GB of RAM, and the Windows 11 operating system.

To improve reproducibility, the main experimental settings used in preprocessing, feature selection, and classification are summarized in Table 7.

## 5. Evaluation Metrics and Results

This section presents the evaluation setup for the proposed model, including the performance metrics and the corresponding experimental results.

### 5.1. Evaluation Metrics

To assess the performance of the model, five standard metrics were computed: false positive rate (FPR), accuracy (Acc), Precision (P), Recall (R), and F1-score. These are derived from the confusion matrix components—true positives (TPs), true negatives (TNs), false positives (FPs), and false negatives (FNs). Such metrics are widely used in IDS evaluation, as they capture both overall classification performance and the ability to correctly detect attacks while minimizing false alarms. The following equations formally define these measures:

(5) $$\begin{array}{cc} \mathrm{FPR} & =\frac{\mathrm{False}\;\mathrm{Positives}}{\mathrm{False}\;\mathrm{Positives}+\mathrm{True}\;\mathrm{Negatives}}\\ \; & =\frac{FP}{FP+TN} \end{array}$$

(6) $$\mathrm{Acc}=\frac{TP+TN}{TP+TN+FP+FN}$$

(7) $$\mathrm{Precision}=\frac{TP}{TP+FP}$$

(8) $$\mathrm{Recall}=\frac{TP}{TP+FN}$$

(9) $$F1-\mathrm{score}=\frac{2\;\mathrm{Precision}\cdot \mathrm{Recall}}{\mathrm{Precision}+\mathrm{Recall}}$$

Finally, the area under the receiver operating characteristic curve (AUC-ROC) is also used to evaluate the classifier. The ROC curve summarizes the trade-off between TPRs and FPRs across different decision thresholds, and the AUC offers a measure of overall discriminative ability that is independent of thresholds.

### 5.2. Experimental Results

We assess the efficiency of our model, ML-FSID-FIS, on the test set employing common measures like accuracy, Precision, Recall, F1-score, FPR, and AUC. The data is divided into three parts in a balanced way: 70% for training, 15% for validation, and 15% for testing. We scale the features using only the training data to make sure there is no data leakage. We note that the present evaluation is based on a single stratified 70%/15%/15% train–validation–test split on the WUSTL-EHMS-2020 dataset. Although this protocol provides a clean separation between training, threshold selection, and final testing, additional validation using repeated random splits, cross-validation, or multiple datasets would further strengthen the evidence for generalization and robustness. In addition, the present study does not yet report repeated-run statistical measures such as standard deviation, confidence intervals, or hypothesis testing. Therefore, the reported improvements should be interpreted within the scope of the current evaluation protocol.

**Figure 6:**
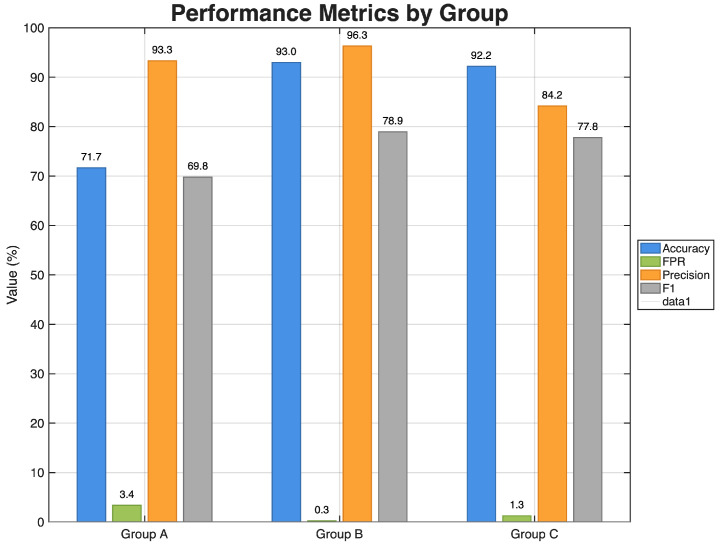
Performance of the FIS model with three 3-input feature groups (A, B, C).

Our main evaluation focuses on the final 3-input FIS configuration derived from the multi-level feature selection phase. To assess the robustness of the model to feature selection, we further evaluate three alternative 3-feature groups—A, B, and C—formed from the top five ranked features. A side-by-side comparison across all metrics is shown in Figure 6 and summarized numerically in Table 8.

Considering all metrics in Figure 6 and Table 8, the three configurations exhibit distinct behaviors. In terms of accuracy, Groups B and C clearly outperform Group A, achieving 93.0% and 92.2%, respectively, compared to 71.7% for Group A.

Precision is another important metric for evaluating the proposed system, as it reflects the proportion of alarms that correspond to actual attacks. High Precision implies that most of the detected intrusions are indeed relevant, which is crucial for reducing the time and effort spent on investigating false alarms. Among the three groups, Group B attains the highest Precision, 96.3%, followed by Group A, 93.3%, and Group C, 84.2%. This shows that the ML-FSID-FIS model, when configured with Group B features, generates highly trustworthy alerts and therefore saves computational and operational resources by minimizing spurious detections.

To further quantify our model performance, the F1-score takes the trade-off between accurately identifying attacks and limiting false positives by integrating Precision and Recall into a single harmonic mean value. Groups B and C once again outperform Group A in terms of F1-score: Group B achieves the maximum F1-score of 78.9%, Group C comes in slightly lower 77–8.0%, while Group A falls short at 69%. Thus, Group B provides a good balance between detecting attacks and avoiding unneeded alerts, as indicated by its combination of high Precision with the best F1-score, while the lower F1-score for Group A indicates that it misses a larger fraction of attacks despite its relatively high Precision.

From the five most important features, we select three possible three-feature sets (A, B, and C). When comparing accuracy and F1-score, Groups B and C are significantly better than Group A. Group B provides the best overall performance, with an accuracy rate of 93.0% and a precision rate of 96.3%, all while keeping the lowest false positive rate (0.3%). The practical deployment of Intrusion Detection Systems in safety-critical IoMT settings is motivated by a low false positive rate (FPR), which indicates that only a tiny percentage of legitimate traffic is incorrectly identified as malicious [31]. As a result, Group B is chosen as the final configuration based on this operating-point performance. In healthcare cyber–physical environments, large numbers of sensors and monitoring devices can generate frequent false alarms, which may confuse operators and reduce the efficiency of clinical workflows; therefore, controlling false-alarm behavior is essential when designing intrusion detection solutions for IoMT deployments [32].

**Figure 7:**
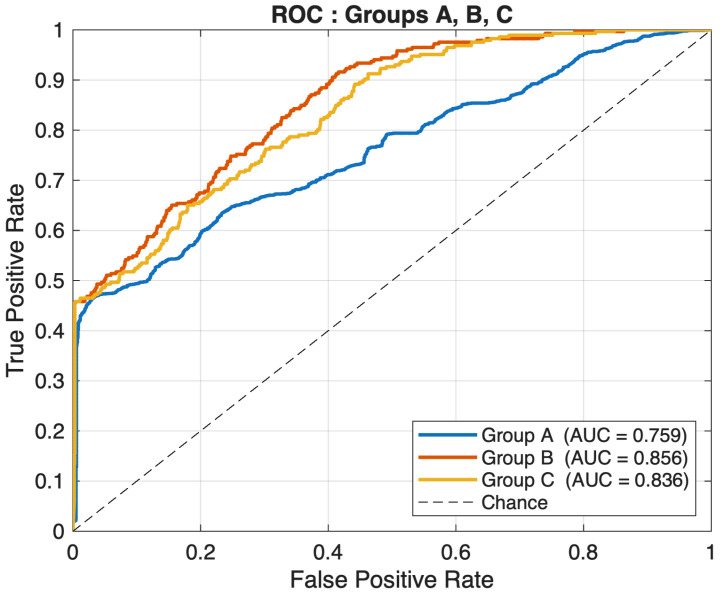
ROC curves for the three feature groups (A, B, C).

Figure 7 further assesses the discriminative capacity of the three groups through ROC curves. Group A attains the lowest area under the curve AUC = 0.759, confirming that this feature combination provides the weakest separation between normal and attack traffic. In contrast, Groups B and C have much higher AUC values at 0.856 and 0.836 respectively, indicating that both configurations consistently rank attack samples above benign ones with greater reliability across various decision thresholds. The ROC curves for Groups B and C are closely aligned, with Group B having a slightly better AUC, but Group C still performs very well in terms of ranking.

**Figure 8:**
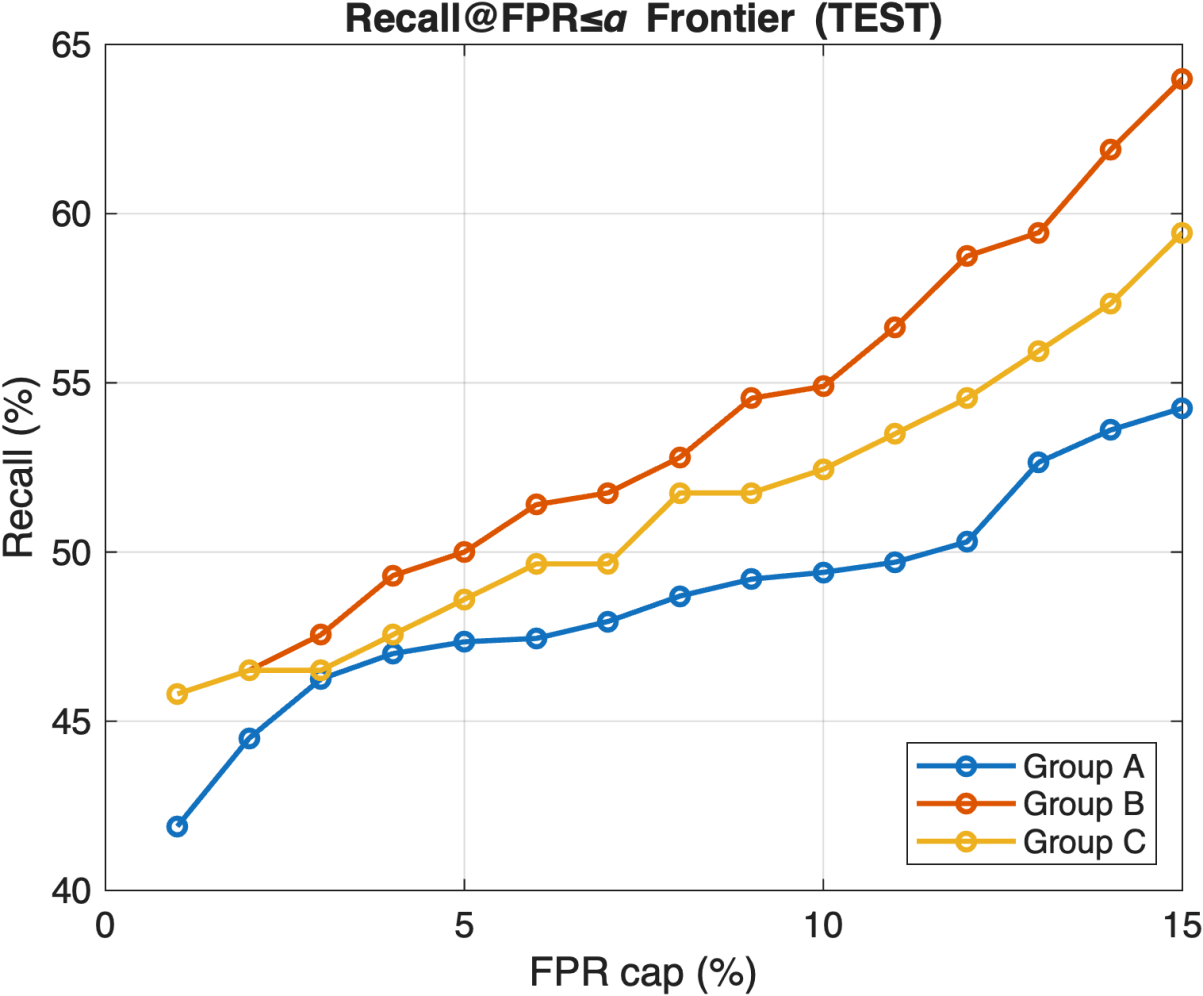
Recall@FPR frontier for feature groups A, B, and C on the test set.

Figure 8 shows Recall as a function of the allowed FPR. For each FPR cap, we choose a threshold on the validation set that satisfies the cap and then report the resulting Recall on the test set. When the FPR budget is increased, the Recall improves for all groups. Across different FPR levels, Group B remains the best performing, with Recall around 46% at 1% FPR, 50% at 5%, and 64% at 15%. Group C is consistently second, showing a Recall of about 46%, 49%, and 60% at those FPR levels. Group A has the lowest Recall, with values around 42%, 47%, and 54% at the same FPR levels. This view complements the ROC analysis: under strict IoMT settings with very low false-alarm budgets, Group B delivers the best detection performance while keeping alerts under control. These results reflect the operating point trade-off of the proposed IDS: stricter false positive control reduces unnecessary alarms, but it can also lower Recall and allow a portion of attacks to remain undetected. In safety-critical IoMT settings, this trade-off is important because excessive false alarms may reduce the practical usability of the IDS.

When we look at the ROC and Recall@FPR results together, they show that our ML-FSID-FIS model has robust discriminative capability across different decision thresholds for both Group B and Group C feature sets. Group B provides the best balance among the evaluated candidate groups under stringent false-alarm budgets, although this operating point still reflects a trade-off between low false positives and attack detection capability.

In the next section, we compare the performance of the chosen configuration (Group B) with some recent methods for detecting intrusions, highlighting its competitive detection accuracy, low false positive rate, and compact feature set.

## 6. Comparison with State-of-the-Art Methods

This section presents the proposed ML-FSID-FIS model within the context of other IoMT IDS studies that have been tested on the WUSTL-EHMS-2020 dataset. A summary of these methodologies is presented in Table 9. Besides accuracy, we also report the false positive rate, as false alarms immediately impact clinician workload and pose possible risks to patient safety. If a study does not mention the FPR or similar metrics, we label those as *NR* (not reported). The false positive rate (FPR) of our model is reported on a held-out test split at a validation-selected threshold, providing an operationally relevant measure rather than accuracy alone. Overall, ML-FSID-FIS has a very low FPR of 0.3%, and it still performs well in terms of accuracy, using only three input features and a simple, easy-to-understand FIS classifier. In contrast, many existing methods rely on larger feature sets, more complex deep architectures, or do not fully document their false alarm behavior. We note that this comparison is intended to provide context relative to recent studies using the same dataset, rather than a fully controlled benchmark comparison. The compared works differ in evaluation protocols, feature set sizes, validation strategies, and model designs. Therefore, the results should be interpreted as an informative reference rather than as a strictly uniform head-to-head comparison. A controlled baseline re-implementation under identical conditions remains an important direction for future work.

The study in [10] reports a false alarm rate of 2.21%. In comparison, our model, ML-FSID-FIS, reduces this value to 0.3%, corresponding to roughly a 7.4× decrease in false alarms. This reduction is particularly relevant in IoMT settings, where each unnecessary alert can add to clinical workload.

The work in [9] reports an accuracy of about 97.6% with a false positive rate of 3.12% using 10-fold cross-validation. Our model achieves a false positive rate of 0.3% on the held-out test split at a validation-selected threshold.

In [11], an accuracy above 90% is obtained with FPR 6.07% using 34 selected features. By contrast, our suggested model attains FPR 0.3% using only three features, highlighting the effectiveness of the proposed feature selection phase and the resulting compact model.

The study in [14] reports high accuracy but an FPR of 10.1% on WUSTL-EHMS-2020. Such a false alarm rate could cause challenges in safety-critical IoMT environments. High accuracy without careful analysis of FPR can mask operational risk; in contrast, our approach explicitly targets low false-alarm behavior by selecting the decision threshold τ on the validation split and reporting the resulting FPR on the held-out test split.

Finally, the study in [13] achieved 97.85% accuracy with a 3.0% false positive rate using 19 selected features. In comparison, our model uses only three features and achieves an FPR of 0.3% on the held-out test split at the validation-selected threshold τ. This provides a favorable trade-off between compactness, interpretability, and false-alarm control for deployment in resource-constrained IoMT environments.

Based on these studies, ML-FSID-FIS appears promising for safety-sensitive and resource-restricted IoMT environments, since it achieved a very low false positive rate while using a compact feature set in the evaluated setting.

## 7. Conclusions

This paper introduced ML-FSID-FIS, a multi-level feature selection and fuzzy inference-based intrusion detection model for IoMT networks. This work was motivated by the seriousness of clinical cyberattacks in healthcare and the need for an intrusion detection approach that remains reliable when IoMT data are noisy or incomplete. We developed a model that uses multiple feature selection techniques to select consistently informative attributes using a frequency-based consensus strategy before classifying traffic using a compact, interpretable zero-order Sugeno fuzzy inference system.

Experiments conducted on the WUSTL-EHMS-2020 dataset show that the proposed model achieves promising results at an operational point appropriate for safety-critical environments. In the final 3-input configuration, ML-FSID-FIS achieves 93.0% accuracy and 96.3% Precision, while sustaining a 0.3% false positive rate at the threshold established during validation. This low false positive operating point is useful for reducing unnecessary alarms in healthcare environments, although it also reflects a trade-off in which Recall is more modest under strict false-alarm constraints. In practical IoMT implementations, when actual attacks are few, reducing unnecessary alerts helps avoid alert fatigue and unneeded responses, allowing clinicians and analysts to focus on truly suspicious activities. In addition, the model is efficient for resource-limited environments: the multi-level feature selection reduces redundancy and yields a small input set, while the rule-based FIS ensures transparent and verified decisions.

For future work, since this study employs a single benchmark dataset and a single stratified train–validation–test split, we will test ML-FSID-FIS on additional IoMT-related datasets and real network traffic to better assess generalization. We will also examine repeated random splits and cross-validation protocols to provide stronger evidence of robustness, conduct controlled baseline re-implementations under identical experimental conditions for fairer comparison, and include repeated-run statistical measures such as standard deviation, confidence intervals, and hypothesis testing to strengthen validation of the reported improvements, in addition to studying concept drift and adaptive attacks while keeping the fuzzy rules compact and interpretable. Overall, ML-FSID-FIS shows that combining multi-level feature selection with fuzzy inference can provide an effective, interpretable IDS design for IoMT networks within the evaluated setting.

## Figures and Tables

**Table 1:** Examples of network flow and biometric features in the WUSTL-EHMS-2020 dataset.

Feature	Brief Description	Feature	Brief Description
**Flow Metrics**			
SrcBytes	Source packet bytes	DstBytes	Destination packet bytes
SrcLoad	Source load (bytes)	DstLoad	Destination load (bytes)
SrcGap	Source packet gap	SIntPkt	Source inter-packet interval
DIntPkt	Destination inter-packet interval	SIntPkAct	Source inter-packet (active)
DIntPkAct	Destination inter-packet (active)	SrcJitter	Source jitter
DstJitter	Destination jitter	sMaxPktSz	Source max packet size
dMaxPktSz	Destination max packet size	sMinPktsz	Source min packet size
Dur	Flow duration	Trans	Aggregated packet count
TotPkts	Total packet count	TotBytes	Total bytes
Loss	Retransmitted/dropped packets	pLoss	% retransmitted/dropped
pSrcLoss	% source retransmitted/dropped	pDstLoss	% destination retransmitted/dropped
Rate	Packets per second	Load	Load
**Bio Metrics**			
Temp	Temperature	SpO2	Peripheral oxygen saturation
Pulse_Rate	Pulse rate	Sys	Systolic blood pressure
DIA	Diastolic blood pressure	Heart_Rate	Heart rate
Resp_Rate	Respiration rate	ST	ECG ST segment

**Table 2:** Features selected after Random Forest top 10.

# Feature Number	Feature
1	DIntPkt
2	DstJitter
3	Packet_num
4	SrcLoad
5	Dur
6	Load
7	SIntPkt
8	Temp
9	Rate
10	SrcJitter

**Table 3:** Features selected after mutual information top 10.

# Feature Number	Feature
1	Packet_num
2	DstJitter
3	DIntPkt
4	Dur
5	SIntPkt
6	DstLoad
7	SrcLoad
8	Rate
9	Load
10	Pulse_Rate

**Table 4:** Features selected after XGBoost top 10.

# Feature Number	Feature
1	DIntPkt
2	DstJitter
3	Rate
4	SpO2
5	Packet_num
6	Load
7	Temp
8	Resp_Rate
9	Pulse_Rate
10	SYS

**Table 5:** Features selected after ReliefF top 10.

# Feature Number	Feature
1	Pulse_Rate
2	Heart_rate
3	SpO2
4	Resp_Rate
5	ST
6	SrcLoad
7	DIA
8	Load
9	Temp
10	DIntPkt

**Table 6:** Top 10 features obtained after frequency-based consensus across the four methods (Random Forest, XGBoost, Mutual Information, ReliefF).

# Feature Number	Feature
1	DIntPkt
2	Load
3	DstJitter
4	Packet_num
5	SrcLoad
6	Rate
7	Pulse_Rate
8	Temp
9	SpO2
10	Dur

**Table 7:** Main experimental settings of the proposed ML-FSID-FIS model.

Setting	Value
Dataset	WUSTL-EHMS-2020
Data split	Stratified split: 70% training, 15% validation, 15% test
Normalization	Min–max scaling to [0, 1]
Balancing method	SMOTE
Stage 1 feature selection methods	Random Forest, XGBoost, ReliefF, Mutual Information
Stage 1 retained features	Top 10 features from each method
Stage 2 method	Frequency-based consensus strategy
Stage 2 retained features	Top 10 consensus features
Stage 3 method	Bagging-based ranking
Stage 3 retained features	Top 5 features
Candidate groups	Three 3-feature groups (A, B, C)
Final selected group	Group B: DstJitter, DIntPkt, SrcLoad
Classifier type	Zero-order Sugeno FIS
Number of FIS inputs	3
Linguistic terms per input	2 (Low, High)
Input membership function	Generalized bell (gbellmf)
Number of fuzzy rules	8
Decision rule	Validation-selected threshold τ

**Table 8:** Test performance of ML-FSID-FIS for three 3-feature groups (A, B, C) at the validation-selected threshold τ.

Group (Features)	Acc	Recall (%)	F1-Score	Precision	FPR	AUC
A: DIntPkt, Packet_num, DstJitter	71.7	42	69.8	93.3	3.4	0.759
B: DstJitter, DIntPkt, SrcLoad	**93.0**	**46**	**78.9**	**96.3**	**0.3**	**0.856**
C: DstJitter, DIntPkt, Load	92.2	46	77.8	84.2	1.3	0.836

**Table 9:** Comparison of the proposed ML-FSID-FIS with state-of-the-art methods on the WUSTL-EHMS-2020 dataset.

Ref.	Year	Balanced	FS Methods	#FS	Method	Acc. ≥ 90%	FPR	Category	Validation
[10]	2024	NR	PSO	NR	DT/RF/SVM/ KNN/ELM/LR	✓	2.21%	IoMT	NR
[9]	2023	✓	None	0	MLP	✓	3.12%	IoMT	✓
[11]	2022	✓	Var.-analysis; DR	34	RF	✓	6.07%	IoMT	✓
[14]	2024	NR	NR	NR	CNN/LSTM/AEs	✓	10.1%	IoMT	✓
[13]	2024	×	RFE	19	DT (RFE)	✓	3.0%	IoMT	✓
ML-FSID-FIS	2026	✓	MI, RF, ReliefF, XGBoost	3	FIS	✓	0.3%	IoMT	✓

Note: NR—not reported.

## Data Availability

Data are available upon request.
